# Feasibility of intensive insulin therapy in a developing country – the Indian experience

**DOI:** 10.1186/1687-9856-2015-S1-O16

**Published:** 2015-04-28

**Authors:** Hemchand K Prasad

**Affiliations:** 1 Mehta Children’s Hospital, Chennai, Tamil Nadu, India

## 

The superiority of the Intensive insulin regimen over the conventional split mix is very well established in Western centres, often considered a prelude to pump therapy. Developing countries find it a challenge to initiate Basal bolus therapy. The main problems include: lack of support systems in school environment to take up the injections and glucose monitoring at school, and difficulties in assessing carbohydrate content of native foods prepared at home (considering the variability in preparation of any given food item). The parental acceptance of five injections a day also remains a challenge. There are numerous extraneous influences and myths in minds of parents that discourage them from accepting this regimen. We present our experience in surmounting these challenges and establishing Intensive insulin therapy as a first line of insulin therapy and also share the advantages in terms of the glycemic control and growth of children on follow-up with us.

